# Design and implementation of an EEG-based recognition mechanism for the openness trait of the Big Five

**DOI:** 10.3389/fnins.2022.926256

**Published:** 2022-09-07

**Authors:** Bingxue Zhang, Yuyang Zhuge, Zhong Yin

**Affiliations:** Department of Optical-Electrical and Computer Engineering, University of Shanghai for Science and Technology, Shanghai, China

**Keywords:** openness trait, the Big Five, task-related power, spiking neural networks, intelligence

## Abstract

The differentiation between the openness and other dimensions of the Big Five personality model indicates that it is necessary to design a specific paradigm as a supplement to the Big Five recognition. The present study examined the relationship between one's openness trait of the Big Five model and the task-related power change of upper alpha band (10–12 Hz). We found that individuals from the high openness group displayed a stronger alpha synchronization over a frontal area in symbolic reasoning task, while the reverse applied in the deductive reasoning task. The results indicated that these two kinds of reasoning tasks could be used as supplement of the Big Five recognition. Besides, we divided one's openness score into three levels and proposed a hybrid-SNN (Spiking Neural Networks)-ANN (Analog Neural Networks) architecture based on EEGNet to recognize one's openness level, named Spike-EEGNet. The recognition accuracy of the two tasks was 90.6 and 92.2%. This result was highly significant for the validation of using a model with hybrid-SNN-ANN architecture for EEG-based openness trait recognition.

## 1. Introduction

### 1.1. Background

In modern personality psychology, the theory of personality traits defines traits as relatively enduring and stable patterns of behavior, thought, and emotion, which influence individuals' behaviors and make their responses consistent with different situations. Over a hundred years of research, psychologists applied factor analysis to personality survey data and revealed five underlying factors: extraversion, neuroticism, conscientiousness, agreeableness, and openness to experience (Costa and McCrae, 1976; McCrae and Costa, 1987; McCrae and John, 1992). These five traits were known as the “Big Five” personality traits. The Big Five personality traits were applied to multiple fields, such as personnel selection (Holland, 1966; Kern et al., 2019) and mental disorder detection (Khan et al., 2005; Cuijpers et al., 2010).

A great deal of previous studies demonstrated that each trait of the Big Five was more or less correlated with emotional reactivity and affected response. For example, individuals scoring high in neuroticism are more likely to experience negative feelings (e.g., upset, depression, anxiety, jealousy) (Dolan, 2006; Hettema et al., 2006) while agreeableness was negatively related to negative affect (Kuppens, 2005; Letzring and Adamcik, 2015). However, inconsistent results were found in the study of the relationship between openness trait and emotional reactivity (Shiota et al., 2006; Letzring and Adamcik, 2015). Differing from other traits of the Big Five, openness was more strongly related to the intellect rather than affect (McCrae and Costa, 1991; Ready and Robinson, 2008). Those scoring high in the openness dimension were intellectually curious, tended to think in symbols and abstractions, and found repetition boring (Venville et al., 2013). Therefore, openness reflected the ability to process abstract and perceptual information, indicating that it had an underlying connection with one's cognitive interest and ability (DeYoung et al., 2005). Agreement on how to interpret and contextualize the openness factor has not yet been reached. Most importantly, openness was the only dimension of the Big Five to be consistently and positively associated with intelligence (DeYoung et al., 2005). In view of the importance and particularity of openness, it has research and practical value to establish a reliable and accurate measurement method for it.

Like other traits of the Big Five, a common-used measure to evaluate one's openness trait is self-report, which is widely based on questionnaires [e.g., the Revised NEO-Personality-Inventory (NEO-PI-R, Costa and McCrae, 2008) the Big-Five Inventory (BFI, Carciofo et al., 2016)], and self-description (either in sentences Fruyt et al., 2004 or single adjectives Goldberg, 1992). These kinds of measures are undoubtedly quick and direct for self-testing. However, research studies showed that as many as three-quarters of the test-takers achieved a different personality type when tested again (Paul, 2010). Respondents tended to produce comparatively higher nonresponse rates and larger measurement errors in response to questions that were seen as intrusive or easily raised concerns about the possible repercussions of disclosing information (Tourangeau and Yan, 2007). The situation would be even worse when it comes to competitive scenes such as personnel selection. For example, applicants may paint them in the best light in order to obtain the position they want. Therefore, the response to self-report questionnaires would be fake and false.

Compared with the self-report methods mentioned above, brain physiological data offers a more credible way to assess one's openness because it is spontaneously formed and thus hardly forged. Although at its seedling stage, efforts have been made to explore the relationship between the openness trait and various physiological data. However, many of the findings were based on costly and immobile brain imaging devices (e.g., functional magnetic resonance imaging DeYoung et al., 2009; Jiang et al., 2018 and magnetic resonance imaging DeYoung et al., 2010; Liu et al., 2013), which were hardly applied into practice. One feasible solution is to use electroencephalograph (EEG), which is wearable, relatively inexpensive, and high-temporal. By reason of the connection between personality traits and emotional experience, most EEG-based studies of the Big Five focused on task-state EEG signals (De Pascalis et al., 2004; Speed et al., 2015; Suzuki et al., 2019; Li et al., 2020a,b). Accordingly, among these works, emotional-related materials (e.g., emotional videos and words) were used as a stimulus for all the Big Five traits (De Pascalis et al., 2004; Speed et al., 2015; Suzuki et al., 2019; Li et al., 2020a,b). However, as mentioned above, openness had its particularity, that is, it was more connected with intelligence and cognitive ability than with emotional factors (DeYoung et al., 2005; Llúıs-Font, 2005), which resulted in inconsistent statistical results in emotional-related studies (De Pascalis et al., 2004; Zhao et al., 2017). Hence, for a reliable and accurate paradigm to detect one's openness, cognitive control paradigms such as go/no-go task (or emotional go/no-go task Meǵıas et al., 2017, considering the emotional influence on openness) might be more suitable (Amodio et al., 2007; Tottenham et al., 2011). Nevertheless, neither emotional-related tasks nor cognitive control tasks could directly reflect the openness trait (including but not limited to imagination, cognitive exploration, and intelligence, especially the tendency to process abstract inputs).

According to the behavioral characterization of openness, it is not hard to find that openness partly reflects the ability and tendency to seek and comprehend complex patterns of abstract information (i.e., individuals with high openness are better at understanding abstract symbols and paintings) (Rawlings, 2000; Feist and Brady, 2004; DeYoung, 2015). Some findings from the neuroscience theory might support this view. The present research study based on personality neuroscience speculated that openness might be related to the dorsolateral prefrontal cortex (DeYoung et al., 2005; Adelstein et al., 2011). This region was thought to be involved in high-level cognitive processes, such as integration, abstraction, and evaluation. Privado et al. (2017) proposed that auditory and visual information processed by temporal and occipital regions might be transferred to the frontal lobes for applying these processes involved with facets belonging to the openness trait, such as imagination or intellectual curiosity. From the perspective of behavioral data, the correlation between the openness dimension of the Big Five and other well-known personality models could also give us some inspiration. For instance, statistical results showed that openness was positively correlated with “intuition”—one type from sensing-intuitive dimension of the Myers-Briggs Type Indicator (MBTI) (McCrae and Costa, 1989; Furnham, 1996). In MBTI model, intuitive individuals corresponding to highly open individuals prefer abstract information (e.g., symbols, metaphors) and underlying patterns (Martin, 2001). In light of the above, it is suggested that abstract visual stimulus, such as abstract symbols, could reflect one's openness from both behavioral data and the neuroscience theory.

### 1.2. Mixed-reasoning task

EEG enjoys its portability and relatively inexpensive cost than other neuroimaging techniques. In this paper, we proposed a mixed-reasoning task, consisting of two counterbalanced intellectual-related tasks—symbolic reasoning task and deductive reasoning task (more like a verbal intelligence task which pays more attention to vocabulary and comprehension), as a more explainable EEG-based assessment of openness. The main reason why we introduced a counterbalanced deductive reasoning task into the experiment was that general intelligence (*G*) was commonly subdivided into fluid intelligence (*Gf*) and crystallized intelligence (*Gc*). Although openness has been shown to be more strongly associated with *Gc* than *Gf*, both of the two facts substantially contributed to one's openness (Ackerman and Heggestad, 1997; Ashton et al., 2000; DeYoung et al., 2005). Generally speaking, symbolic reasoning tasks were widely used to measure fluid cognitive ability.

The symbolic reasoning tasks aimed to examine an individual's sensitivity to symbolic and abstract stimulus while the deductive one focused on one's capacity to capture details and facts. We chose Raven's Advanced Progressive Matrices (RAPM, Raven and Court, 1938) as the symbolic stimulus source and Law School Admission Test (LSAT)'s logical reasoning questions as the deductive reasoning source.

Raven's Advanced Progressive Matrices asked participants to think logically, based on the rules associated with the symbols in the matrix diagram. RAPM was often used to assess thinking ability, observational ability, and symbolic perceiving ability. Using RAPM as a stimulus can prompt subjects to undertake symbolic reasoning, thus stimulating brain perception (Zhang et al., 2021). The LSAT Logical Reasoning section asked subjects to choose the right answer about logical relationships after reading and comprehending the given passages. This section aimed to examine the subject's ability to analyze logic from literal description. Given the reason that the questions were based on brief arguments drawn from a wide variety of daily sources (e.g., newspapers, magazines, publications, and advertisements), applying the LSAT Logical Reasoning section to deductive reasoning could minimize the influence of the specialized knowledge.

### 1.3. Task-related power of EEG alpha band

More demanding tasks at hand were accompanied by increases in the power of the alpha band (8-13 Hz) (hereinafter referred to as alpha synchronization) which were interpreted as a kind of top-down control that spontaneously happened in mental activities, including attention distribution and memory formation (Cooper et al., 2003; Fink et al., 2006; Benedek et al., 2011; Jaarsveld et al., 2015). The research studies on both verbal (Fink and Neubauer, 2006) and non-verbal (Jaarsveld et al., 2015) intellectual-related-tasks also revealed the relationship between TRP of alpha band and human intelligence. It was worth noting that, besides human intelligence, TRP of alpha band was also speculated to reflect cognitive flexibility—the ability to break conventional rules and experiences (Fink et al., 2006), which was, to a great extent, conformed to the characteristics of openness. It indicated that TRP of alpha band (especially for the intellectual-related tasks) might index one's openness. So far, although abundant works reported the relationship between TRP of alpha band and human intelligence or creativity, very few studies have applied such a conclusion to analyze the underlying relationship between alpha power and openness trait.

Specifically, the alpha band is usually subdivided into lower (8–10 Hz) and upper (10–12 Hz) alpha bands. The TRP in the upper alpha band was more sensitive to our tasks, as the tasks were more associated with imagination and reasoning (Niedermeyer and da Silva, 2005), while the lower band was thought to reflect attention mobilization (Klimesch, 1999). Hence, only the upper alpha band was considered in this paper.

### 1.4. Classification of one's openness

In this paper, one's openness trait was divided into three levels (see Section 2), and a deep learning model was proposed. Over the past years, models based on convolutional neural networks have shown outstanding performance in capturing the underlying complex features of EEG signals. Especially, Lawhern et al. proposed a compact CNN-based model named EEGNet and proved its generalization ability under different BCI paradigms (e.g., sensorimotor rhythm, motor imagery) (Lawhern et al., 2018). EEGNet could directly learn from the original EEG signals without extracting features. However, ANN architecture (e.g., EEGNet) belonged to the second generation neural network, which meant the neurons “spiked” at a fixed frequency. This was not consistent with the real mechanism of the human brain. In light of this, the model using a learning method could encode EEG signals into precisely timed spike trains, which might better learn from the temporal and spatial variations of data.

A spiking neural network (SNN) represents information as binary events (spikes) (Zhan et al., 2021), and thus is thought to correspond more to the biological neuron model. The neurons of SNN fire when the membrane potential accumulates to a set firing threshold. Therefore, SNN provides a promising capacity to model complex information processing in the brain (Beyeler et al., 2013; Kulkarni and Rajendran, 2018; Zhan et al., 2021). Recent studies have proved that SNN had a better performance in multiple EEG tasks, such as brain disease diagnosis (Ghosh-Dastidar and Adeli, 2007; Capecci et al., 2015; Doborjeh et al., 2015), motor imagery signal classification (Carino-Escobar et al., 2016; Mashford et al., 2017; Niranjani and Sivachitra, 2017), and P300 signal classification (Goel et al., 2006, 2008).

However, the limitation of the SNN-based model for EEG tasks was that pure SNN architecture was time-consuming to be trained because the update of weights was based on unsupervised learning rules (e.g., Caporale et al., 2008) in most of the previous studies. On the other hand, the number of spikes were found to decrease rapidly in deep layers (Srinivasan et al., 2020). This made a deep SNN architecture hard to converge (an SNN architecture distinguished different categories by output spikes, so inadequate spikes were not significant to distinguish different categories). In view of the above, we proposed a hybrid-SNN-ANN model based on EEGNet, named Spike-EEGNet in this paper.

### 1.5. Hypotheses

All in all, it was expected that openness would be contextualized by the mixed-reasoning task, through both EEG signals and behavioral data. Specifically, we hypothesized that openness was associated with two variables—accuracy and TRP of alpha band. We assumed that openness would be positively correlated with the performance of two reasoning tasks (symbolic and deductive). Regarding the physiological signals, we hypothesized that higher openness would result in stronger alpha synchronization in both tasks. Concerning the classification model, we expected that a hybrid-SNN-ANN architecture would have a better performance than pure ANN architecture, without sacrificing much time.

## 2. Methods

### 2.1. Label one's openness

The Chinese Big Five Personality Inventory brief version (CBF-PI-B, Wang et al., 2010) was used to measure participants' openness. The questionnaire was a 6-point Likert scale including 40 items, 8 measures for each trait. The internal consistency coefficients (Cronbach's alpha: extraversion = 0.80, openness = 0.78, neuroticism = 0.81, conscientiousness = 0.81, and agreeableness = 0.76) and the factors correlated with relevant dimensions of NEO-PI-R (extraversion = 0.76, openness = 0.66, neuroticism = 0.74, conscientiousness = 0.85, and agreeableness = 0.36) both indicated that CBF-PI-B had good psychometric properties, which could be adopted in relevant research (Wang et al., 2010).

Most previous studies on EEG-based assessment of personality were mainly based on binary classification and reported a substantial accuracy (Farnadi et al., 2016; Zhao et al., 2017). A binary classification meant, in short, that all individuals scoring below the threshold were classified as a low type and those scoring above the threshold were classified as the opposite type. However, this kind of classification was limited to its statistical meaning. A mountain of evidence has revealed that the overall scores of personality scales were actually distributed in a centrally peaked manner, which was more similar to a normal distribution than a bimodal distribution. It indicated that the majority of people actually lay near the middle of the scale (Howard and Howard, 1995; Bess and Harvey, 2002; Chamorro-Premuzic et al., 2005; Fleeson and Gallagher, 2009), rather than near both end of the scale.

**Figure 1:**
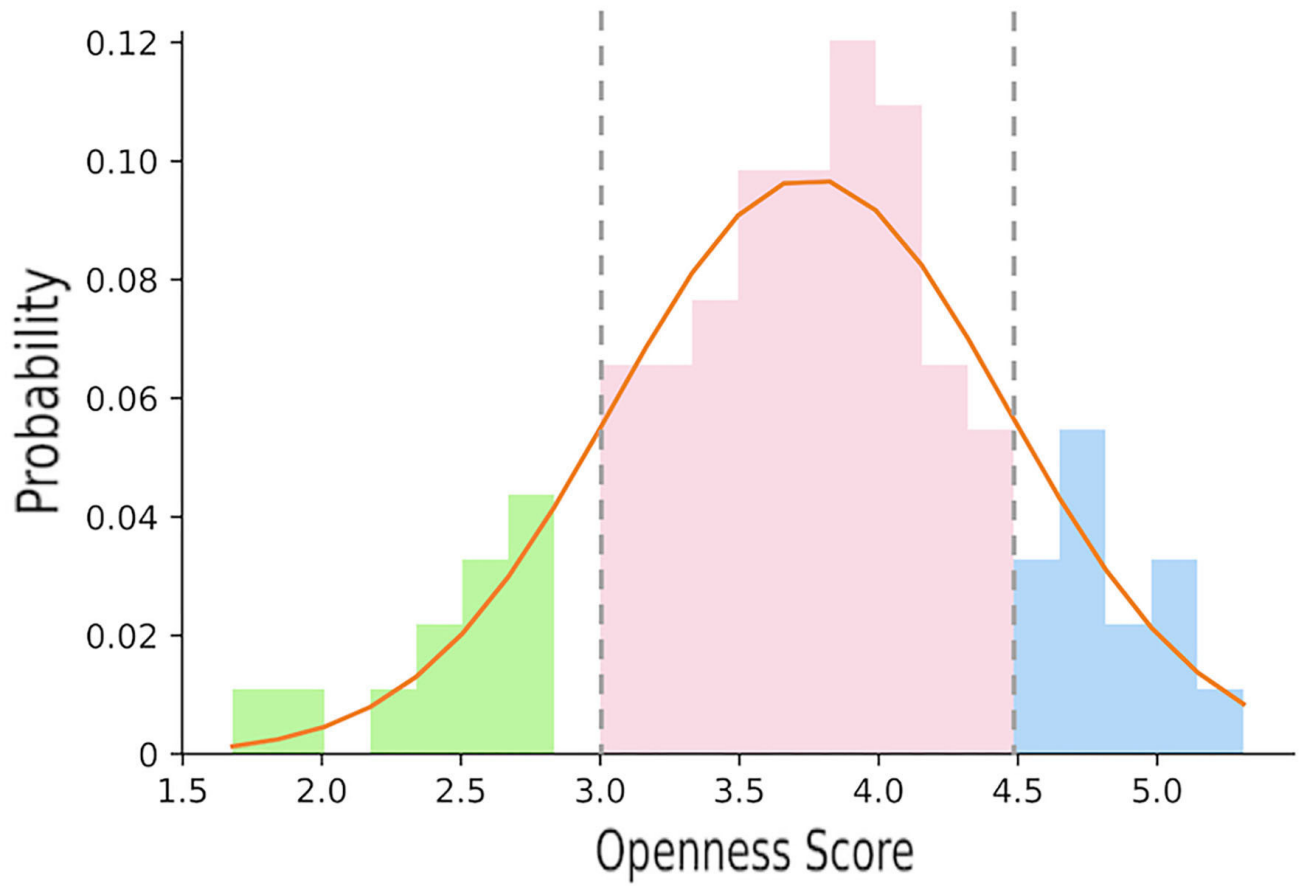
The distribution of participant's openness score.

In this work, the openness recognition task was defined as a ternary classification task. In view of a normal distribution of scores on all the Big Five dimensions (Howard and Howard, 1995; Fleeson and Gallagher, 2009), we made a division based on the mean scores (μ) and standard deviation (σ) for the openness dimension of all the participants: scoring below μ−σ as low openness, between μ−σ and μ+σ as medium openness, above μ+σ as high openness. As shown in Figure 1, a gap was observed between the score in the range (2.8, μ−σ), meaning that the participants scoring below μ−σ could be regarded as a “cluster.” Besides, the distribution of scores in range (μ−σ, μ+σ) could be regarded as the main modality of the whole distribution (Shapiro-Wilk Test, *p*>0.05) and the rest could be thought of as the minor modality. In conclusion, defining the problem as a ternary classification task was relatively reasonable.

### 2.2. Participants

CBF-PI-B questionnaires were distributed to 100 college students from the University of Shanghai for Science and Technology, and 95 were collected. These 95 students were then divided into three groups according to their levels of openness, using the method we mentioned above. For each level of openness, 8 students voluntarily participanted in further experiments. Thus, this study examined 24 healthy volunteer participants (12 male; age range = 20−31 years, mean age = 24.42 years). All participants were native Chinese speakers, right-handed, with normal vision or corrected to normal. All were free of neurological and psychiatric disorders. No subjects had taken medicines and no habitual drinkers or smokers. Written informed consent was obtained from each participant before the investigation. Each participant was paid a small fee for participating in the study.

### 2.3. Task

Participants were asked to complete the symbolic reasoning task and deductive reasoning task in turn. In each task, the estimated completion time was limited to 40 min in order to reduce fatigue and cognitive load caused by lengthy and single reasoning (Wascher et al., 2016). Thus, four blocks of 6 trials were conducted for both tasks.

As discussed above, RAPM and LSAT Logical Reasoning questions were applied to the symbolic reasoning task and deductive reasoning task separately. In each trial of the symbolic reasoning task, participants were asked to fill in vacant positions using the appropriate options. What is noteworthy is that only Sections *D* and *E* of RAPM were chosen [RAPM has 60 questions, which are divided into five equal sections (Sections *A*, *B*, *C*, *D*, *E*), with increasing difficulty]. Because there are 8 options for each question from Section *D* to *E*, compared with only 5 choices for LSAT Logical Reasoning questions, three wrong choices for each RAPM questions were removed to ensure that the expected value of the right choice in both tasks was equal.

In each trial of the deductive reasoning task, participants were asked to choose the best answer from 5 choices about the facts mentioned in or inferred from the given passage. All questions were translated to the subject's common language.

### 2.4. Procedure

Before the experiment, participants sat comfortably in an electrically shielded room, approximately 100 cm from a computer screen. They were instructed about the rules and the meaning of the symbols in the tasks and given one practice trial for each task.

During the formal tasks, each block began with the appearance of an instruction related to the task type on the screen. Participants were asked to press “*Enter*” until they fully read and comprehend the instruction. After a 10-s fixed cross presentation at the center of the screen, the question and choices were presented on the screen.

In the deductive reasoning task, passages were presented at the upper center and followed by five vertically-presented choices. In the symbolic reasoning task, symbolic patterns were presented at the upper center and five laterally-presented choices were beneath it. Participants were asked to make a response by pressing the “1” or “2” or “3” or “4” or “5” key consistent with the given choices on the keyboard with their left or right index finger after they fully understood and reflected. In other words, the reasoning time was not limited, thus eliminating the mental workload caused by the time limitation. The question and choices remained visible on the screen for 500 ms after the participant pressed the button, and then were replaced by the feedback, which was presented at the center of the screen for 1,000 ms. After a 5-s fixed cross, the subsequent trial began.

**Figure 2:**
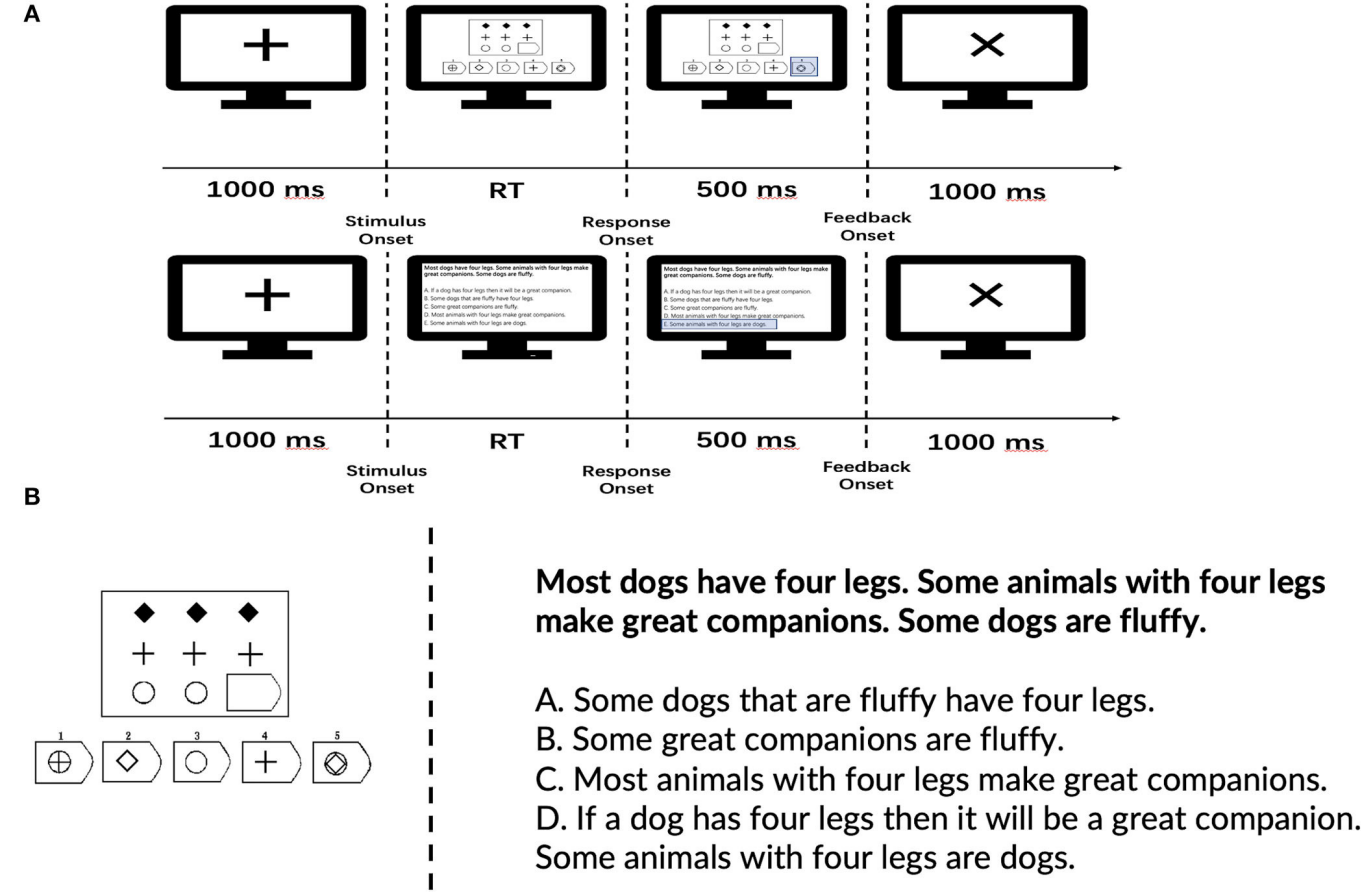
**(A)** Experimental procedure of the two tasks. RT denotes reaction time, **(B)** examples according to the type of reasoning task—symbolic reasoning on the left-hand side and deductive reasoning on the right-hand side.

The procedure and example trials of the two tasks are shown in Figure 2. Question display and behavioral data acquisition were conducted using E-Prime 2.0 (Psychology Software Tools, Inc., Pittsburgh, PA, USA). Participants were asked to take a break between the blocks.

### 2.5. EEG recordings

The Emotiv Epoc+ (Emotiv Systems, San Francisco, USA) was used for EEG recordings. The Emotiv Epoc+ was a wireless headset with flexible plastic arms that held 14 AgCl/Ag sensors placed at AF3, F7, F3, FC5, T7, P7, O1, O2, P8, T8, FC6, F4, F8, and AF4 (plus CMS/DRL references, P3/P4 locations), according to the International 10–20 system. The signals were high-pass filtered at 0.16 Hz and pre-amplified using a low-pass filter at 83 Hz. The EEG was then downsampled to 128 Hz, using the Emotiv Xavier TestBench software (Emotiv Systems, San Francisco, USA). All sensors were adjusted until the software control panel showed “green” before the onset of the experiment, which represented that the electrode impedance required by the software was reached (in the 10–20 *kΩ* range).

### 2.6. Synchronizing the EEG and behavioral data

Because the Emotiv systems do not have its own synchronizing equipment, a Python script using a Python package named pynput (https://github.com/mosespalmer/pynput) was written to synchronize the EEG and behavioral data. Pynput created its own thread to record keyboard and mouse events in millisecond Unix time. In order to reduce the possibilities of serial port conflicts, TestBench software for EEG recording and E-Prime 2.0 for behavioral data were run parallelly on two computers. The two same scripts were run on these two computers separately. Both of the computers were networked to proofread time in real time. After pressing a button or clicking to start EEG recording, the time *t*_1_ was recorded. Similarly, after pressing a button or clicking to start the experiment, the time *t*_2_ was recorded. The time difference Δ*t* between two systems could be calculated as *t*_2_−*t*_1_. Thus, if a time stamp recorded by E-Prime was *T* millisecond after onset, its real time stamp should be *T*+Δ*t*. The script was set to terminate automatically after capturing the specific events in order to reduce memory load.

### 2.7. EEG data preprocessing and quantification of task-related alpha power

EEG data analysis was performed using the EEGLAB toolbox (Delorme and Makeig, 2004) running under Matlab 7.8.0 (The Mathworks Inc.). The EEG data was bandpass filtered from 0.1 to 50 Hz using a linear finite impluse response (FIR) filter. The order and transition bandwidth was set to 9 and 0.35 Hz, respectively. The processed EEG signals were then filtered for the upper alpha frequency band (10 to 12 Hz) by employing a fast Fourier transform (FFT) filter applied to Hanning time windows of 1,000 ms with 900 ms overlap. Power estimates were obtained by squaring filtered EEG signals, and then band power values (μ*V*^2^) were averaged for each single trial (Jaarsveld et al., 2015). The TRP changes of single trial for each electrode position *i* in the alpha band were quantified according to the formula:


(1) $$\begin{array}{r} TRP\left(i\right)=log\left[Pow_{i,activation}\right]-log\left[Pow_{i,reference}\right] \end{array}$$

by following the method of Pfurtscheller (1999). For each trial, the 5-s time interval during the presentation of the fixation cross was served as a reference interval, and the activation interval was defined as 1-s time interval before a response was made. For both the reference and activation intervals, EEG data were carefully checked for artifacts, and artifactual epochs caused by muscle tension, eye blinks, or eye movements were excluded from further analysis. For each task, the TRP changes of the alpha band for each participant were averaged to minimize noise. Thus, a positive value of TRP represented the alpha synchronization from reference interval to activation interval while a negative value reflected power decrease (hereinafter referred to as alpha desynchronization).

### 2.8. Statistics

For behavioral data (response time and accuracy), a two-way repeated-measures analysis of variance (ANOVA) was performed, with openness level (low *vs*. medium *vs*. high) as between-subject factors and type of task (symbolic reasoning *vs*. deductive reasoning) as within-subject factors.

Regarding TRP measurements, a two-way repeated-measures analysis of variance (ANOVA) was performed separately on each task, with openness level (low *vs*. medium *vs*. high) as between-subject factors and brain areas [AF_3, 4_ (anterior frontal), F_3, 4_, F_7, 8_ (frontal), FC_5, 6_ (fronto central), T_7, 8_ (temporal), P_7, 8_ (parietal), and O_1, 2_ (occipital), where odd and even numbers indicate the left and right hemisphere, respectively] as within-subject factors. *Post-hoc* analysis of the interaction was computed with Tukey HSD multi-comparison. Pearson correlation coefficients were calculated when appropriate. The alpha level of significance was set at 0.05 throughout the process. Significantly, in RAPM, one participant was excluded for EEG analysis in each group, while two participants were excluded in LSAT, due to excessive artifacts and/or noise caused by long wear of the Emotiv Epoc+ (the saline injected into the sensors might run off). Thus, 7 participants for each level of openness in RAPM and 6 in LSAT were used for analysis.

### 2.9. Spike-EEGNet

The original EEGNet consists of five blocks (input, 2−*D* convolution, depthwise 2−*D* convolution, separable 2−*D* convolution, and classification block). In this paper, we only transformed the neurons in the 2−*D* convolution block into IF neurons, in order to ensure the spikes propagate to deeper layers. The preprocessed data of one activation interval (1-s time under 128 Hz, with 14 channels) was defined as the input. The input was firstly scaled to [−1, 1] across channels, and then fed into the Poisson generator for *T* time steps. Specifically, at each time step, each scaling data entered was compared with the generated random number. If the scaled data were greater than the random number, an output spike was fired. The plus-minus sign of the scaled data was also reserved for the output spike. The output spike was then fed into the 2−*D* convolution block with IF neurons. The membrane potential *V* of the IF neurons in 2−*D* convolution block was updated using the following formula:


(2) $$\begin{array}{r} V\left[t+1\right]=V\left[t\right]+w*o\left[t\right] \end{array}$$

where *w* represented the synaptic weights of 2−*D* convolution block, *o*[*t*] represented the output spike generated from the Poisson generator at discrete timestep *t*. Once the membrane potential reached the set threshold *V*_*th*_, it emitted an output spike and reset to zero. The algorithm was repeated over all the timesteps, and the membrane potential after the last timestep was fed into the following blocks, as the original EEGNet did. It was noteworthy that because the spike train generated from IF neurons was discontinuous, the gradients of the IF neurons were approximated as $\frac{1}{V_{th}}$, based on the method of Lee et al. (2020). The forward and backward propagation algorithm of Spike-EEGNet is described in Algorithm 1.

**>Algorithm 1:** Forward and backward propagation of Spike-EEGNet for an Iteration.


**Require:** Mini-batch of scaled input (*X*) - target (*Y*) pairs, total number of discrete time-steps (*T*), synaptic weights of 2-D convolution block with IF neurons(*w*_*S*_), outputs in SNN block (*o*_*S*_), ANN outputs (*o*_*A*_), membrane potential(*V*), firing threshold (*V*_*th*_), other ANN blocks nonlinearity(*f*), wights of other ANN blocks (*w*_*A*_), loss fuction (*Loss*)
1: Initialize *V*[*t*] = 0
2: // Forward propagation of 2-D convolution block with IF neurons
3: **for** *t* = 1 to *T* **do**
4: *o*_*S*_[*t*] = *PoissonGenerator*(*X*)
5: *V*[*t*] = *V*[*t*−1]+w*o_*s*_[*t*]
6: if *V*[*t*]>*V*_*th*_ then
7: *o*_*S*_[*t*] = 1; *V*[*t*] = 0
8: **end for**
9: // Forward propagation of other blocks
10: *o*_*A*_ = *f*(*w*_A_*V[*T*])
11: // Backward propagation of other blocks
12: $\Delta w_{A}=\frac{\partial Loss}{\partial o_{A}}\frac{\partial o_{A}}{\partial w_{A}}$
13: // Backward propagation of 2-D convolution block with IF neurons
14: $\Delta w_{S}=\frac{\partial Loss}{\partial o_{S}\left[T\right]}\frac{\partial o_{S}\left[T\right]}{\partial V\left[T\right]}\frac{\partial V\left[T\right]}{\partial w_{S}}$, where $\frac{\partial o_{S}\left[T\right]}{\partial V\left[T\right]}=\frac{1}{V_{th}}$

In RAPM, 504 samples were available for training and testing (7 participants in each group, 24 time-intervals for each participant), while 432 in LSAT (6 participants in each group, 24 time-intervals for each participant). For each task, 80% of samples were split for training, while 20% for testing. Accuracy, precision, recall, and F1 score were chosen as the criteria to evaluate the performance of the model. The standard results were averaged after 5-fold cross validation.

The architecture of Spike-EEGNet is shown in Table 1. The firing threshold *V*_*th*_and timestep *T* were set to 2 and 16 in both datasets.

**Table 1:** Spike-EEGNet architecture.

**Block**	**Layer**	**Numbers of filters**	**size of filter**	**Output**	**Remarks**
1	Input			(14,128)	
	Poisson generator			(14,128)	
2	2*D* convolution with IF neurons	8	(1,64)	(8,14,128)	Padding = same
	BatchNorm			(8,14,128)	
3	Depthwise 2*D* convolution	16	(14,1)	(16,1,128)	Padding = valid
	ELU activation			(16,1,128)	
	Average pooling		(1,4)	(16,1,32)	
	Droupout			(16,1,32)	*p* = 0.5
4	Separable 2*D* convolution	16	(1,16)	(16,1,32)	Padding = same
	BatchNorm			(16,1,32)	
	ELU activation			(16,1,32)	
	Average pooling		(1,8)	(16,1,4)	
	Droupout			(16,1,4)	*p* = 0.5
5	View			(64)	
	Dense			(3)	
	Softmax			(3)	

## 3. Result

### 3.1. Self-reported openness score

The distribution of 95 individual scores is shown in Figure 1. Shapiro-Wilk Test was applied before further analysis in order to guarantee the normality assumption (*p*>0.05). There was no significant difference between the scores of male participants and those of female participants. The mean scores and SD for the openness traits dimension were 3.75 and 0.71, respectively. Therefore, according to our classification, individuals with low openness were quantified as those scoring below 3.04, while high openness individuals obtained scores over 4.46.

### 3.2. Accuracy

**Figure 3:**
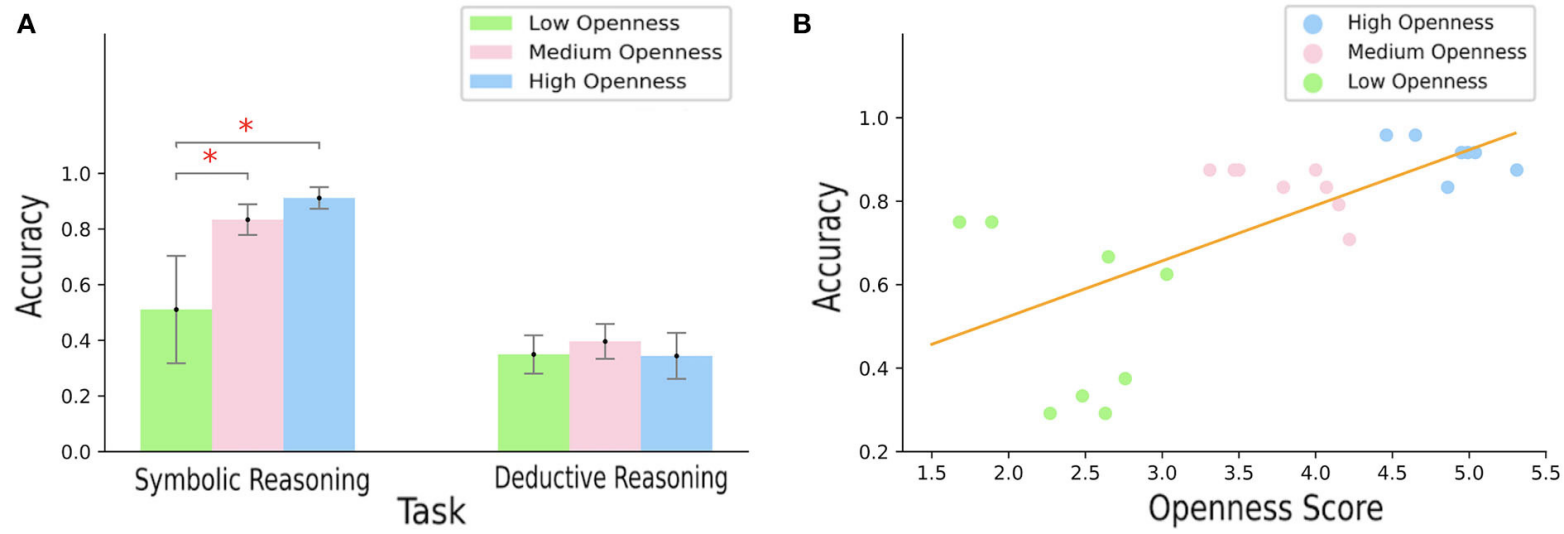
**(A)** Participants' accuracy according to the type of reasoning task—symbolic reasoning on the left-hand side and deductive reasoning on the right-hand side, **(B)** Pearson's correlation scores computed between the openness scores and accuracy in the deductive task, according to the openness level—low, medium, and high openness corresponding respectively to green, pink, and blue bars/dots.

As shown in Figure 3A, for medium and high openness, higher accuracy was obtained in symbolic reasoning tasks than in deductive reasoning tasks. In symbolic reasoning task, accuracy appeared to be positively correlated to openness. In addition, no significant difference could be visualized for deductive reasoning tasks. Statistical comparisons supported these observations. The two-way repeated measures ANOVA revealed significant main effect of openness [*F*_(2,42)_ = 14.67, *p* < 0.0001], significant main effect of task [*F*_(1,42)_ = 187.20, *p* < 0.0001], and significant interaction [*F*_(2,42)_ = 11.94, *p* < 0.0001]. *Post-hoc* analysis of interaction showed higher accuracy in medium and high openness groups than in low openness group in symbolic reasoning task (both *p* < 0.001), and higher accuracy in symbolic reasoning task than in deductive reasoning task in both medium and high levels of openness (both *p* < 0.001). Further, regarding the symbolic reasoning task, a significant strong correlation between the openness score and accuracy was found (Pearson's *r* = 0.45, *p* < 0.05, Figure 3B). Results showed there was no significant difference among openness levels in deductive reasoning tasks.

### 3.3. Reaction times

**Figure 4:**
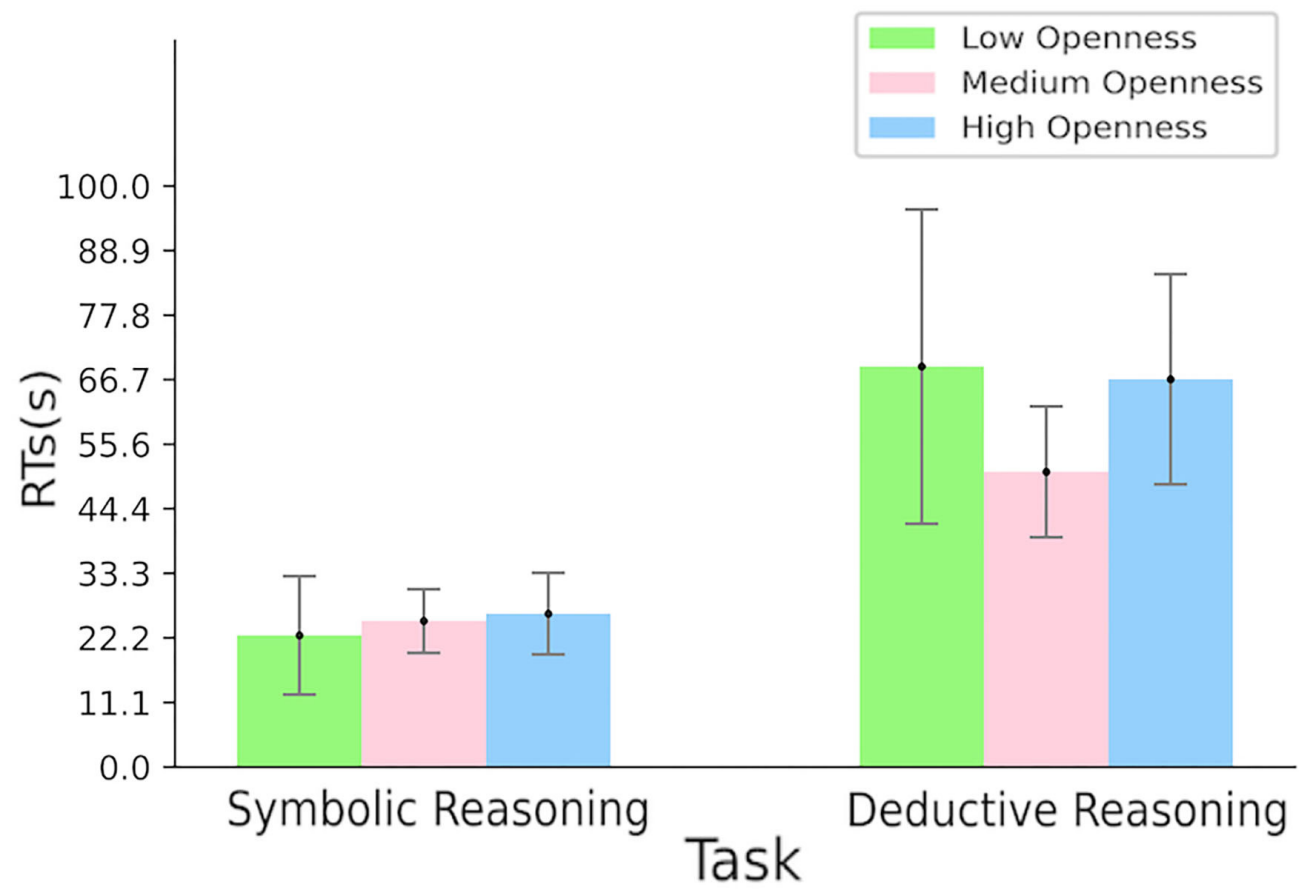
Participants' RTs according to the type of reasoning task—symbolic reasoning on the left-hand side and deductive reasoning on the right-hand side, according to the openness level—low, medium, and high openness corresponding, respectively, to green, pink, and blue bars.

Regarding RTs, the two-way repeated measures ANOVA revealed a significant main effect of task [*F*_(1,42)_ = 64.46, *p* < 0.0001, Figure 4], indicating that deductive reasoning tasks cost more time than the symbolic one. Although in the deductive reasoning task, the RTs of high and low openness groups seemed to be higher than those of the medium openness group, no significance was found.

### 3.4. TRP of the alpha band in RAPM

**Figure 5:**
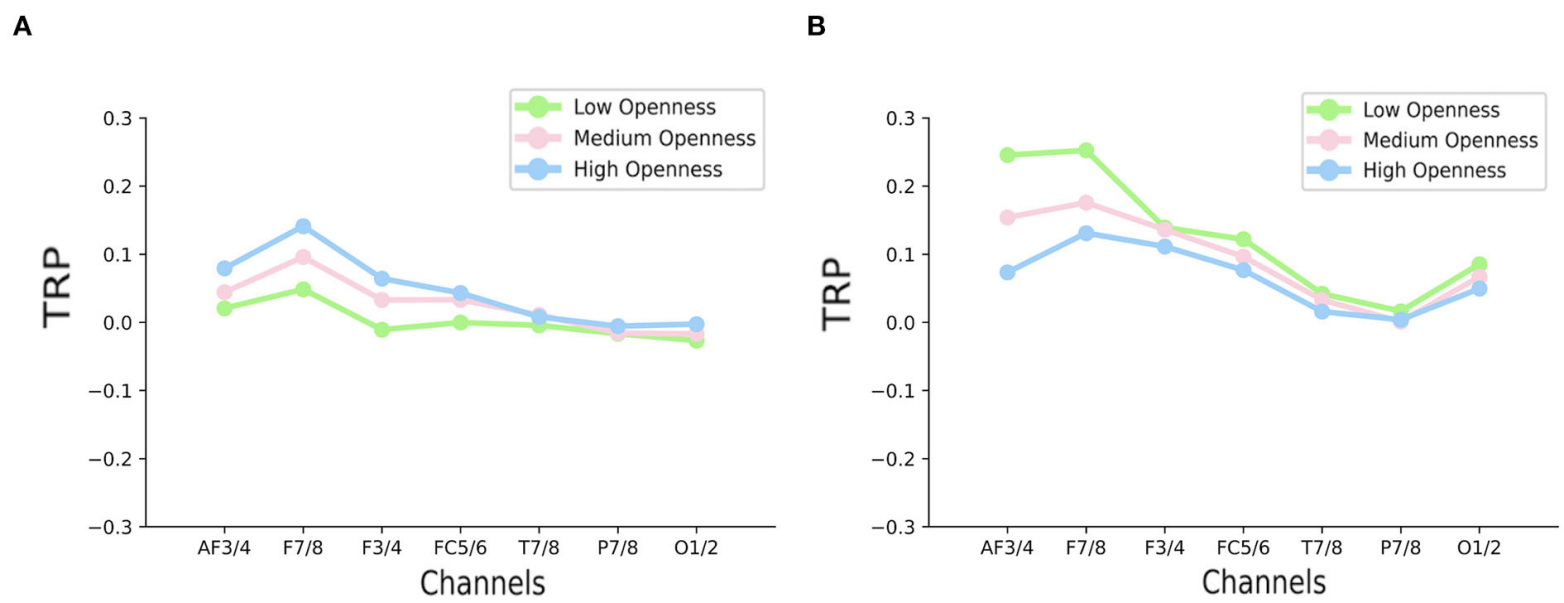
Task-related power (TRP) changes in the upper alpha band over seven channel pairs, **(A)** in RAPM, **(B)** in LSAT, according to the openness level—low, medium, and high openness corresponding, respectively, to green, pink, and blue dots.

Figure 5A shows the TRP of the alpha band at 7 brain areas in RAPM. We could observe that compared with the other two groups, the alpha synchronization of the high openness group was significantly stronger, mainly over frontal areas (F7/8, F3/4 electrodes).

Statistical comparisons on TRP supported these observations. The two-way repeated measures ANOVA revealed a significant main effect of openness level [*F*_(2,126)_ = 5.41, *p* < 0.01] and a significant main effect of brain area [*F*_(6,126)_ = 4.87, *p* < 0.01]. No significant interaction was found. *Post-hoc* analysis of main effects showed that the difference in TRP between openness levels was significant over F7/8, F3/4 (both *p* < 0.05). No significance was found in other areas.

### 3.5. TRP of the alpha band in LSAT

Figure 5B shows the TRP of the alpha band at 7 brain areas in LSAT. In contrast to the RAPM, we could observe markedly stronger alpha synchronization in the low openness group than the other two groups mainly over anterior frontal and frontal areas (AF3/4, F7/8 electrodes).

Statistical comparisons on TRP supported these observations. The two-way repeated measures ANOVA revealed a significant main effect of openness level [*F*_(2,105)_ = 9.12, *p* < 0.01] and a significant main effect of brain area [*F*_(6,105)_ = 8.91, *p* < 0.01]. No significant interaction was found. *Post-hoc* analysis of main effects showed that the difference in TRP between openness levels was significant over AF3/4, F7/8 (both *p* < 0.05). No significance was found in other areas.

### 3.6. Classification performance

**Figure 6:**
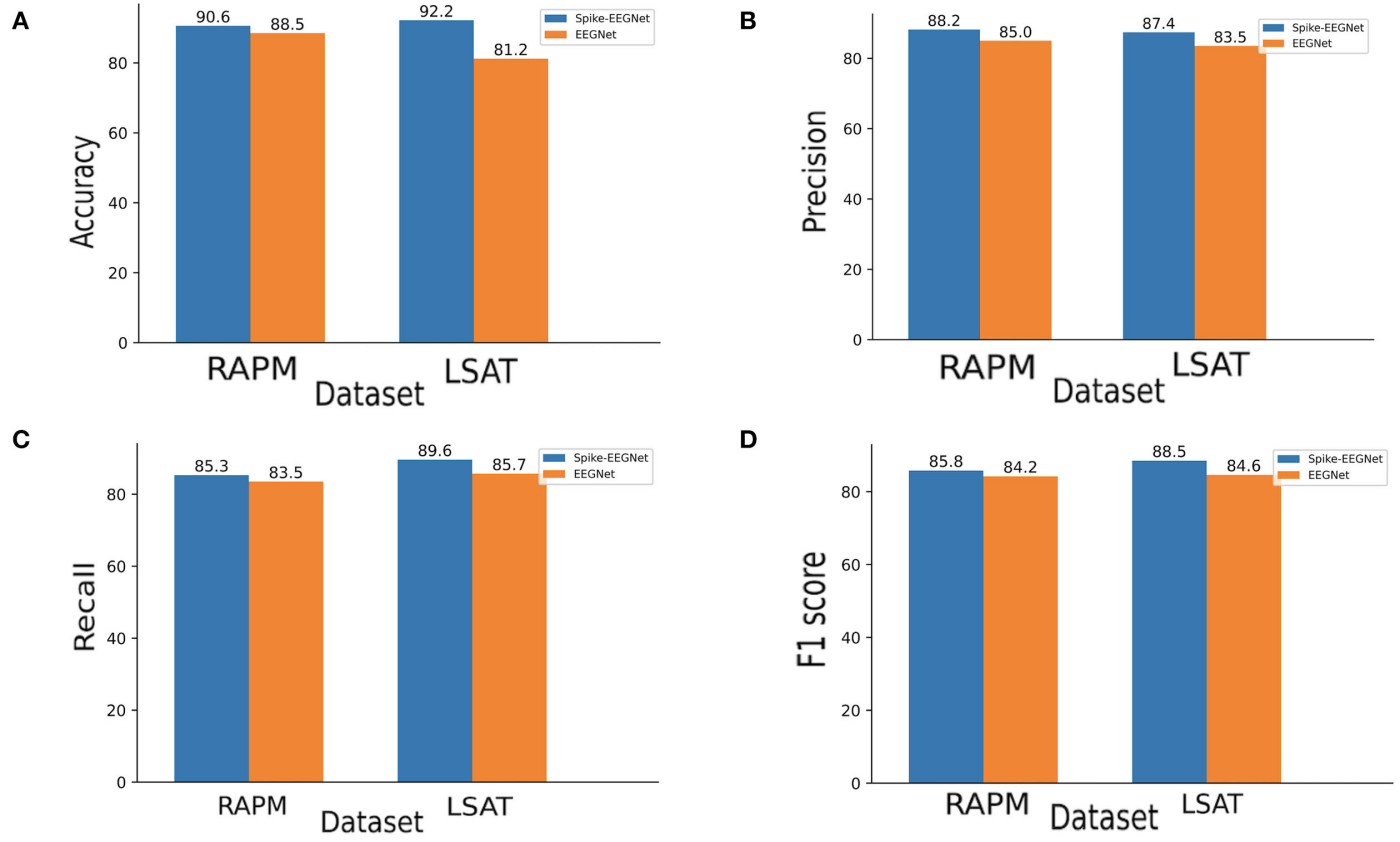
The performance of Spike-EEGNet and the originial EEGNet on two datasets, **(A)** accuracy, **(B)** precision, **(C)** recall, and **(D)** F1 score—Spike-EEGNet and the originial EEGNet corresponding respectively to dark blue and orange histogram.

**Figure 7:**
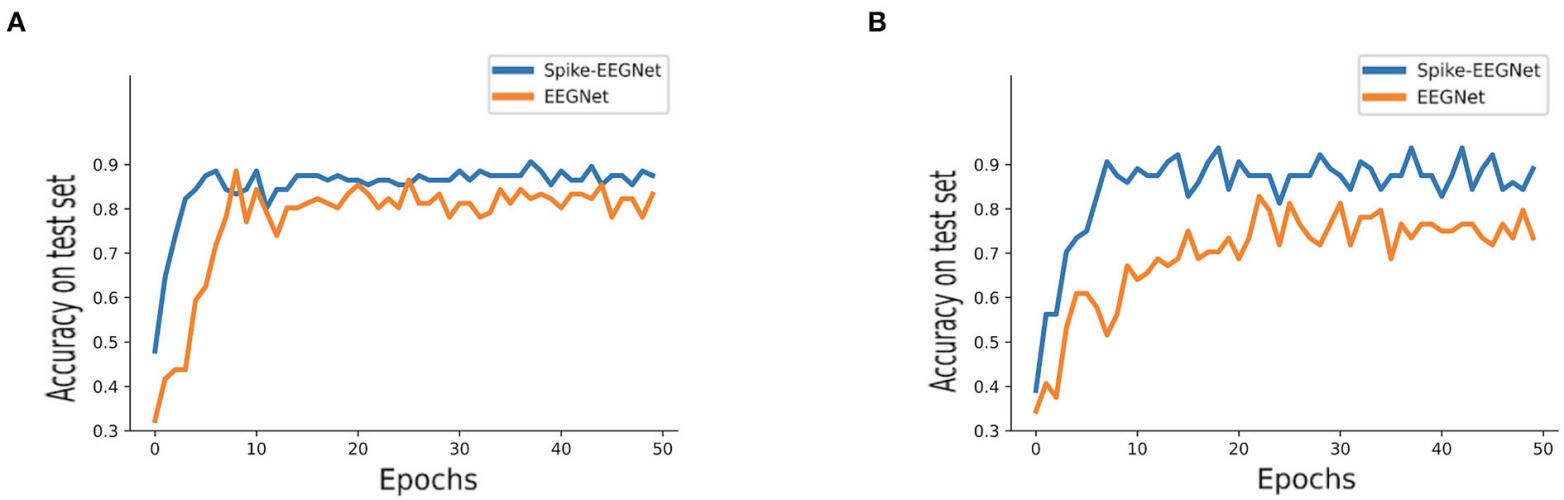
The training process of Spike-EEGNet and the originial EEGNet, **(A)** on RAPM dataset, **(B)** on LSAT dataset—Spike-EEGNet and the originial EEGNet corresponding, respectively, to dark blue and orange broken line.

We compared the performance of original EEGNet and Spike-EEGNet on our two datasets separately. Figure 6 shows the results of the original EEGNet and Spike-EEGNet on accuracy, precision, recall, and F1 score. It could be seen that, in RAPM dataset, Spike-EEGNet achieved recognition accuracy of 90.6%, a precision of 88.2%, a recall of 85.3%, and an F1 score of 85.8%. Meanwhile, Spike-EEGNet achieved a recognition accuracy of 92.2%, a precision of 87.4%, a recall of 89.6%, and an F1 score of 88.5%. Spike-EEGNet achieved a better performance on all criteria. Figure 7 shows the training process of two models on two datasets.

## 4. Discussion

In this paper, we expected openness could be contextualized by behavioral performance and EEG signals in two kinds of reasoning tasks. We assumed that openness would be positively correlated with accuracy and RTs. We hypothesized that higher openness would result in stronger alpha synchronization in both tasks.

As we expected, the accuracy of the RAPM task was generally correlated to one's openness, at least measuring by the O scale of CBF-PI-B. The result was consistent with those of previous studies (DeYoung et al., 2005; Voronin et al., 2016). The only point unlike the previous studies using NEO-PI scale, openness was more modestly associated with RAPM (the Pearson's r between openness scale and RAPM was round 0.3, *p* < 0.01) in this work. This might be ascribed to the otherness of different scales. However, with regard to the accuracy in deductive reasoning, no significant difference was found among levels of openness. We suggested that it might be attributed to the difficulty of the LSAT. This could be also evident from the significant difference between the RTs of the two tasks. Another possible reason was that the deductive reasoning task, which was designed to evaluate one's *Gc*, was more or less contaminated by *Gf*. After all, it was a reasoning-based comprehensive task. Even so, the significant difference between the accuracy of the two tasks in high and medium openness groups indicated that their being sensitive to cognitive ability (fluid vs. crystallized) was different. Regarding the RTs in each task, in agreement with previous studies, openness did not correlate with the overall computational speed (Bates and Shieles, 2003; Voronin et al., 2016). This indicated that levels of openness might not substantially influence an individual's attention and concentration during relatively long-time reasoning.

In both reasoning tasks, the difference in alpha synchronization among openness levels was mainly showed in (anterior) frontal areas. Thus, the following discussion was restricted in these areas. In RAPM, our results supported the position that higher openness groups displayed stronger alpha synchronization in certain areas, presumably indicated that higher openness individuals had more original ideas in capturing the underlying patterns between geometric components, based on the theory of Jaarsveld et al. (2015). In LSAT, however, the reverse applied. For the low openness group, this could be interpreted as a reduced excitability level of neurons in verbal understanding than in symbolic understanding, while in high openness group the opposite was found (Jung-Beeman et al., 2004). Such opposite results were also found among typical divergent thinking tasks (usually involving high internal processing demands, e.g., LSAT) and typical convergent thinking tasks (often involving higher bottom-up processing, e.g., RAPM) (Benedek et al., 2011; Jaarsveld et al., 2015). In light of this, during deductive reasoning, low openness individuals presumably had better internal processes of retrieval and recombination of semantic associations of the stimulus concept, thereby reducing the need for further bottom-up processing of the stimulus (Benedek et al., 2011).

Concerning the classification model, as we expected, a hybrid-SNN-ANN architecture could improve the performance of the pure ANN architecture. Surprisingly, Spike-EEGNet reached a stable accuracy faster than the original EEGNet. It is noteworthy that the hybrid-SNN-ANN architecture will be more energy-consuming than the pure ANN one, due to the reasoning phase among the given timesteps. The reasoning phase is nonparallel. It was suggested to reduce the timesteps if necessary. In this work, the timestep was set to 16 throughout. A longer reasoning timestep might improve the performance because the initial spike generated from the Poisson generator would be more approximate to the true distribution.

## 5. Conclusion

In this paper, we proposed a mixed-reasoning-task to index one's openness trait of the Big Five. RAPM was applied to the symbolic reasoning task, and LSAT was applied to the deductive reasoning task. We found that individuals from the high openness group displayed a stronger alpha synchronization over the frontal area in the symbolic reasoning task, while the reverse applied in the deductive reasoning task. The results indicated that the differentiation of individuals' openness trait could reflect in alpha synchronization of the frontal area in these two kinds of reasoning tasks.

We proposed a hybrid-SNN-ANN model named Spike-EEGNet, based on the original EEGNet. In both datasets, Spike-EEGNet had a better performance. This result was highly significant for the further exploration of using hybrid-SNN-ANN architecture for EEG tasks.

## Data availability statement

The datasets presented in this study can be found in online repositories. The names of the repository/repositories and accession number(s) can be found in the article/supplementary material.

## Ethics statement

The studies involving human participants were reviewed and approved by University of Shanghai for Science and Technology. The patients/participants provided their written informed consent to participate in this study. Written informed consent was obtained from the individual(s) for the publication of any potentially identifiable images or data included in this article.

## Author contributions

BZ: conception and design of study. YZ: analysis and/or interpretation of data and drafting the manuscript. ZY: revising the manuscript critically for important intellectual content. All authors contributed to the article and approved the submitted version.

## Funding

We thank the support of the National Natural Science Foundation of China (62007024) and the Shanghai Sailing Program (17YF1428400). The National Natural Science Foundation of China (62007024) supports us to buy the EEG apparatus for conducting experiments and the Shanghai Sailing Program (17YF1428400) supports us to pay the open access publication fees.

## Conflict of interest

The authors declare that the research was conducted in the absence of any commercial or financial relationships that could be construed as a potential conflict of interest.

## Publisher's note

All claims expressed in this article are solely those of the authors and do not necessarily represent those of their affiliated organizations, or those of the publisher, the editors and the reviewers. Any product that may be evaluated in this article, or claim that may be made by its manufacturer, is not guaranteed or endorsed by the publisher.
